# Adaptive phase contrast microscopy to compensate for the meniscus effect

**DOI:** 10.1038/s41598-023-32917-6

**Published:** 2023-04-08

**Authors:** Florian Nienhaus, Tobias Piotrowski, Bastian Nießing, Niels König, Robert H. Schmitt

**Affiliations:** 1 Fraunhofer Institute for Production Technology IPT, Aachen, Germany; 2 WZL | RWTH Aachen University, Aachen, Germany

**Keywords:** Biomedical engineering, Mechanical engineering, Engineering, Optics and photonics, Optical techniques

## Abstract

Phase contrast is one of the most important microscopic methods for making visible transparent, unstained cells. Cell cultures are often cultivated in microtiter plates, consisting of several cylindrical wells. The surface tension of the culture medium forms a liquid lens within the well, causing phase contrast conditions to fail in the more curved edge areas, preventing cell observation. Adaptive phase contrast microscopy is a method to strongly increase the observable area by optically compensating for the meniscus effect. The microscope’s condenser annulus is replaced by a transmissive LCD to allow dynamic changes. A deformable, liquid-filled prism is placed in the illumination path. The prism’s surface angle is adaptively inclined to refract transmitted light so that the tangential angle of the liquid lens can be compensated. Besides the observation of the phase contrast image, a beam splitter allows to simultaneously view condenser annulus and phase ring displacement. Algorithms analyze the displacement to dynamically adjust the LCD and prism to guarantee phase contrast conditions. Experiments show a significant increase in observable area, especially for small well sizes. For 96-well-plates, more than twelve times the area can be examined under phase contrast conditions instead of standard phase contrast microscopy.

## Introduction

Phase contrast microscopy first proposed in 1932 by Frits Zernike^1^ is a widely used method to observe biological samples because it can make transparent, unstained cells visible^2^. Due to interference, phase shifts of passing light can be made visible to increase contrast in semi-transparent objects.

However, its applications are limited by the meniscus effect, which particularly affects samples in microplates with 96 or more wells^3^. Reference measurements have shown that in 6-well-plates, phase contrast conditions can be found in 25% (235 mm^2^ of 950 mm^2^) of the well surface area. In 96-well-plates, it is only 2.3% (0.84 mm^2^ of 36.3 mm^2^)^4^.

### Phase contrast microscopy

**Figure 1:**
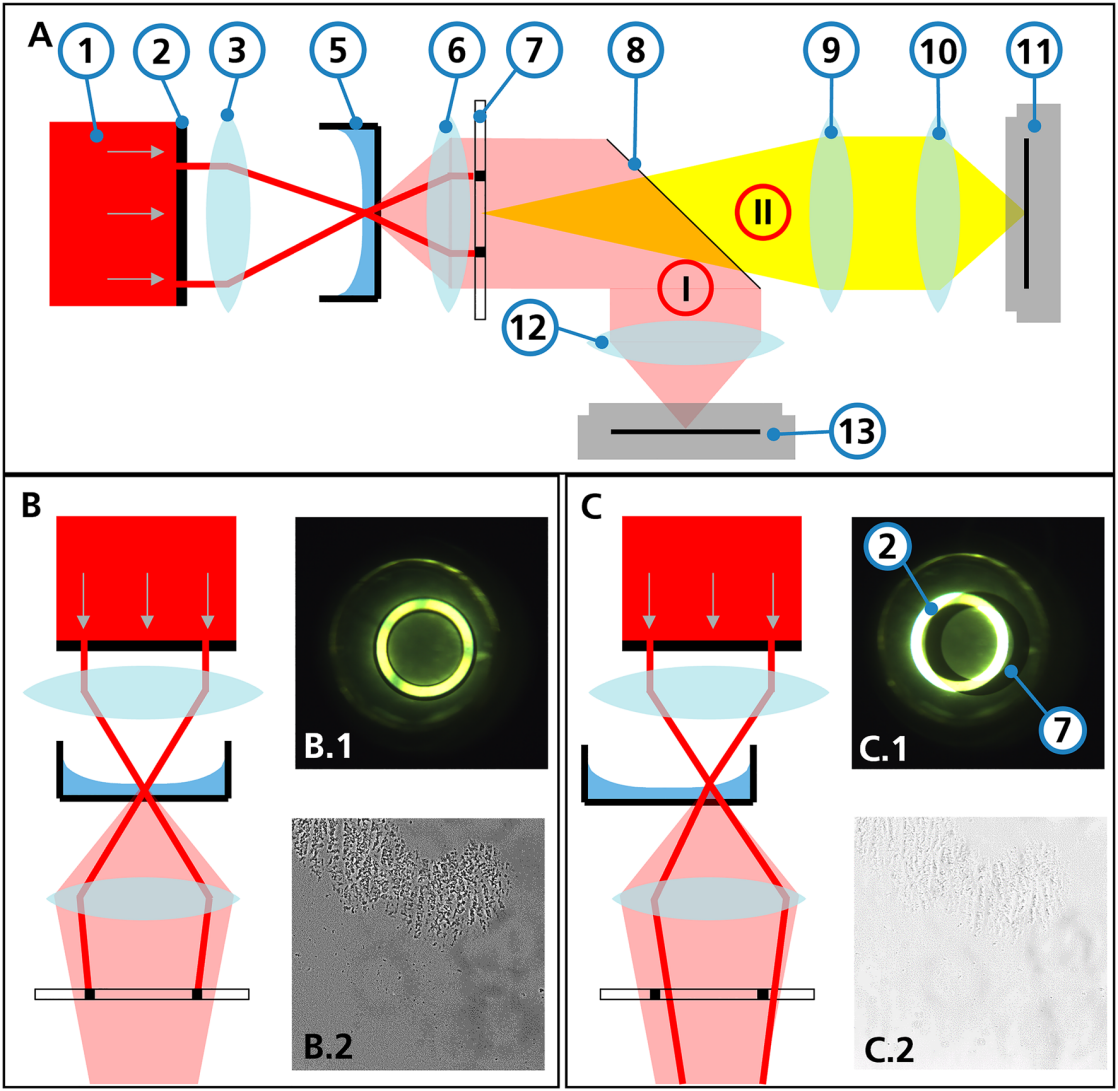
Schematic representation of the illumination path in a phase contrast microscope with different well positions. (**A**) Simplified light path through the microscope (non-active elements such as deflecting mirrors and glass plates have been omitted). (1) Light source (2) Condenser annulus (3) Condenser lens (5) MTP (6) Objective lens (7) Phase ring (8) Movable mirror (9) Bertrand lens (10) Ocular lens (11) Secondary camera (12) Tube lens (13) Main camera. By moving the mirror (8), it can be switched between the main light path (I) and the secondary light path (II) to observe the phase ring and condenser annulus overlap. (**B**) Light passes through the center of the well. (**B.1**) Superposition image of phase ring and condenser annulus. They completely overlap. (**B.2**) Resulting phase contrast image of cell culture. (**C**) Due to the curvature of the surface towards the edge of the well, the light beam is refracted away from the center. (**C.1**) In the superposition image, the deviation between the condenser annulus (2) and the phase ring (7) is visible. (**C.2**) The resulting cell image is thus taken under brightfield conditions. Images (**A.2**) and (**B.2**) were taken with a 10x Objective (Nikon CFI Plan Fluor DL 10XF) in a 6-well MTP.

Phase contrast is a transmitted light microscopy method in which a condenser annulus is placed in the illumination beam path, and the light in the objective is guided through a phase ring (see Fig. 1). If the condenser annulus image and phase ring overlap, phase contrast conditions arise. Phase shifts occur at transitions in the observed specimen, such as cell boundaries, which are optically highlighted. Phase contrast conditions can be easily identified by the "halo" effect, which is a region with a dark background and bright edges around phase shifting objects^2,6^.

Biological samples are usually in a liquid medium. A flat liquid surface is essential for a good superposition of the condenser annulus and phase ring, as seen in Fig. 1B,C. This superposition of condenser annulus and phase ring can be observed via a Bertrand lens unit and a secondary camera.

### The meniscus effect

Cells are often cultivated in standardized vessels such as microtiter plates (MTP), which consist of multiple wells. The surface curvature of the culture medium in each well creates a liquid lens due to capillary forces on the vessel wall, as shown in Fig. 1. The curved surface deflects the incoming light and therefore prevents a superposition of the condenser annulus and phase ring. The phase contrast fails particularly in the more strongly curved edge areas of this lens, thus cells in these areas cannot be imaged^5–7^.

The exact shape of the meniscus can be approximated by numerically integrating the Young–Laplace equation^8^. The smaller the diameter of a well, the higher the influence of the capillary forces at the wall becomes relatively due to the increasing circumference-to-surface area ratio. Consequently, the proportion of the flat liquid surface in the center of the vessel decreases^3^. The greater curvature along the well’s edges results in a larger refraction angle, which in turn increases the distortion and displacements of the condenser annulus light that prevents ring superposition.

### Objectives

Adaptive phase contrast microscopy aims to strongly increase the observable area by optically compensating for the meniscus effect. This setup’s goal is to increase the phase contrast area of a well to a value close to the physical limit, which is set by the surface angle for total reflection^9^. The solution shall be a compact device that can be added to a commercially available microscope without much preparation. Moreover, standard microplates shall be used instead of special plates introduced in “Competing approaches”^12^. Full automation of the process is desired to image whole wells without human interference. Therefore, this paper introduces a fully automated approach that can be included in commercially available standard microscopes.

### Competing approaches

Due to the importance of phase contrast microscope images of cells in microtiter plates, various solutions have been developed in the past that address the problem of the meniscus effect. Those approaches can generally be grouped into the following categories: preventing a meniscus from forming, optically correcting the meniscus effects in the MTPs, reducing the effect on images computationally, and microscopes that optically compensate for the meniscus effect. The setup presented in this paper belongs to the last category.

Possibilities to prevent the meniscus from forming range from hydrophobically coated microplates to lids that push down the solvent’s surface so that it is flat. Instead of direct modifications of the microtiter plate, plastic inserts for use in individual standard wells exist^10–12^.

Another application are MTPs with lids that are shaped such that they work as a prism themselves and thus compensate for the diffraction caused by the meniscus effect. Those lids, however, only work for one specific cell medium with a defined refractive index^13,14^.

Besides special hardware, computational approaches for meniscus effect compensation also exist, such as enhancing contrast by comparing several images, for instance at different z-planes^15^. Other methods use bright-field images, which are modified by artificial intelligence algorithms to produce phase-contrast-like images^16,17^. Those images, however, do not reach the same quality as real phase contrast images.

Finally, there are approaches to compensate the meniscus effect in the optical path of the microscope, like the one proposed in this paper. One suggested solution is to adaptively deform and displace a virtual condenser annulus in accordance to the liquid surface so that after passing through the meniscus it is refracted such that it resembles an undistorted ring again. To create the virtual condenser annulus, a liquid crystal display (LCD) is used^18^. This aspect, namely the displacement of the condenser annulus shape, is also presented in this paper. However, the basic principle explained by this paper additionally uses a deformable liquid prism for this purpose. It is protected by European patent EP 3 323 010 B1 which is the property of Fraunhofer Gesellschaft^19^.

Another approach is to integrate a spatial light modulator into the beam path instead of the LCD. In particular, reflective spatial light modulators have very high pixel density and contrast ratio. However, their integration is much more complex, since the chip size is usually much smaller than the condenser annuli to be displayed. Transmissive spatial light modulators also have a small active area, even though further possibilities exist for distorting the condenser annulus. A digital mirror device can also be used instead of a spatial light modulator^20^.

Yet another approach is to mechanically move the condenser annulus horizontally. This assures the same high contrast and light intensity as regular phase contrast microscopy. However, it is slow and requires complex mechanics.

## Methods

To prove the concept of adaptive phase contrast microscopy, a demonstrator has been built that includes the main components, an LCD condenser annulus, and a liquid-filled prism. It was then used for experiments to compare regular with adaptive phase contrast microscopy. The most important details of the setup are presented below.

### Setup

**Figure 2:**
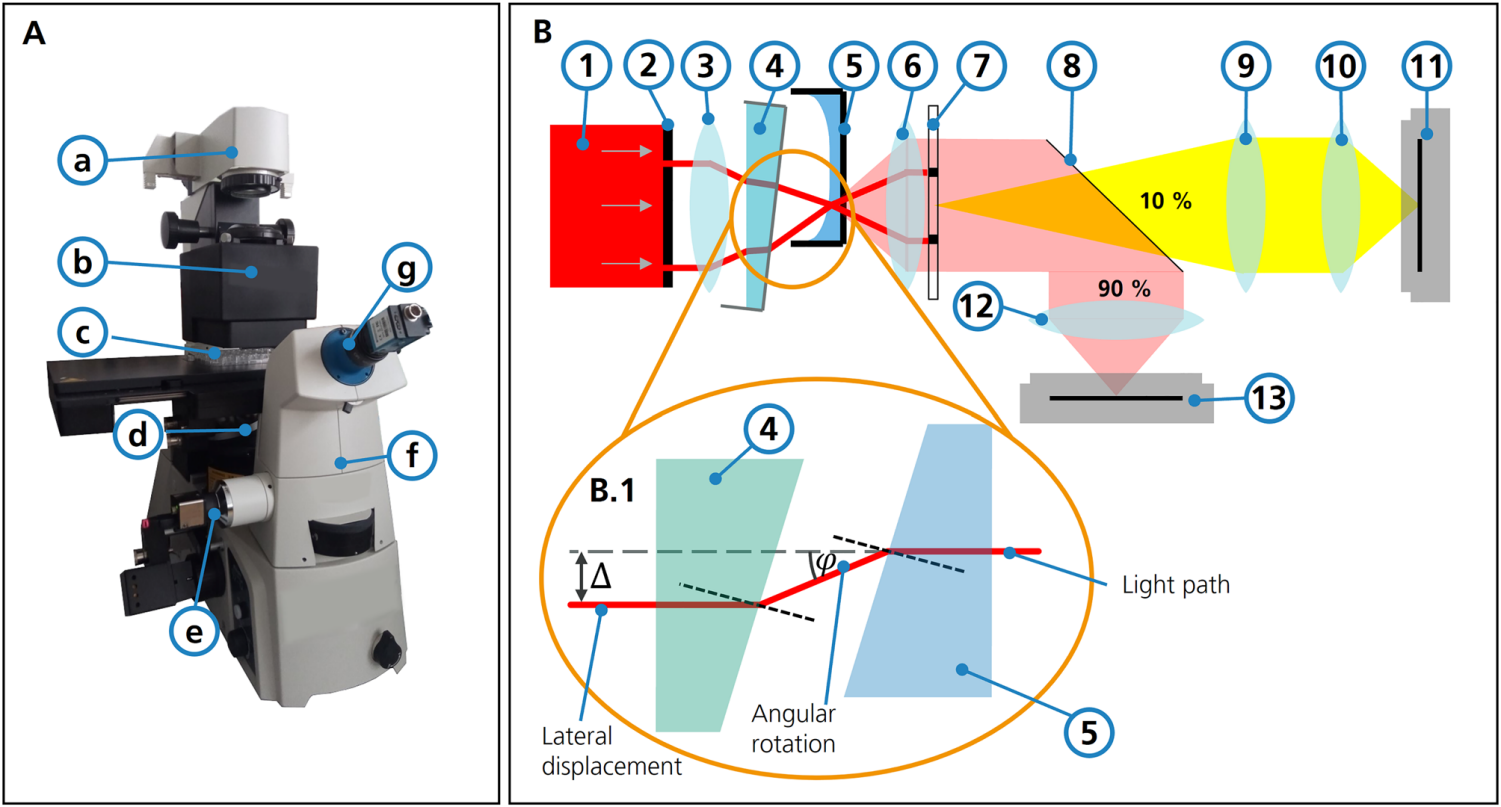
Setup of modified microscope and simplified light path. (**A**) Setup of the elements within an inverse microscope. (a) Light source (b) Optical components adapter (c) MTP (d) Objective lens (e) Main camera (f) Beam splitter (g) Bertrand lens and secondary camera (**B**) Modified light path compared to the one presented in Fig. 1. The fixed condenser annulus (2) has been replaced by a transmissive LCD. A tunable prism (4) is introduced. The movable mirror (8) is replaced by a 90/10 beam splitter. (**B.1**) Working principle of the compensation considering refraction on liquid surfaces (same refractive index in prism and cell medium assumed).

Optical elements are inserted into the beam path of an inverted microscope (Ti2 Eclipse, Nikon, Japan). A compensation adapter replaces the condenser annulus unit in the illumination beam path. Figure 2 shows an overview of the setup and the schematic light path.

The light from the light source (LED100, Märzhäuser Wetzlar, Germany) is guided through a transmissive LCD (LS055R1SX04, Sharp, Japan), the condenser (ELWD, Nikon, Japan), an adaptable prism, the sample within an MTP, and the phase contrast objective with integrated phase ring (4x magnification: CFI Plan Fluor DL 4XF, Nikon, Japan; 10x magnification: CFI Plan Fluor DL 10XF, Nikon, Japan). Thereafter, it passes through the beam path of the microscope body. The deflection mirror in front of the tube lens is replaced by a 90/10 beam splitter (BS028—90:10, Thorlabs, USA). 90% of the light is guided through an ocular lens to the main camera (UI-3360CP-NIR-GL, IDS, Germany) focusing the cells in the MTP. 10% are used for display of overlapping condenser annulus and phase ring through the standard Bertrand lens unit of the microscope and a secondary camera (DFK 33UX265, The Imaging Source, Germany).

The microscope is equipped with a motorized XY stage (SCANplus IM, Märzhäuser Wetzlar, Germany). Since a whole MTP well cannot be covered with just one image, multiple images are taken and then stitched to one image according to their position^21^.

The compensation adapter presented in Fig. 2B.1 is composed of an LCD, a condenser lens, and an adaptable prism. The LCD generates a virtual condenser annulus black-and-white matrix, which can be dynamically adjusted in position and shape to compensate for lateral displacement $\Delta$. The adaptive liquid-filled prism is inserted into the illumination between the condenser and sample to compensate the tangential angle $\varphi$ of the liquid lens.

### LCD

The LCD^22^ can display the condenser annulus as a light–dark matrix. It allows Ø15.5 mm apertures and a shift of the ring center of 5 mm in XY-direction.

The display backlight illumination was removed and replaced by an external LED to achieve higher brightness. This was done because high contrast and high pixel density improve the displayed condenser annulus. Two displays with different pixel densities, 533 ppi (LS055R1SX04, Sharp, Japan) and 187 ppi (CP11009, QITA, United Kingdom), were tested. It turned out that a higher pixel density is necessary for an accurate representation of condenser annulus.

### Adaptive prism

**Figure 3:**
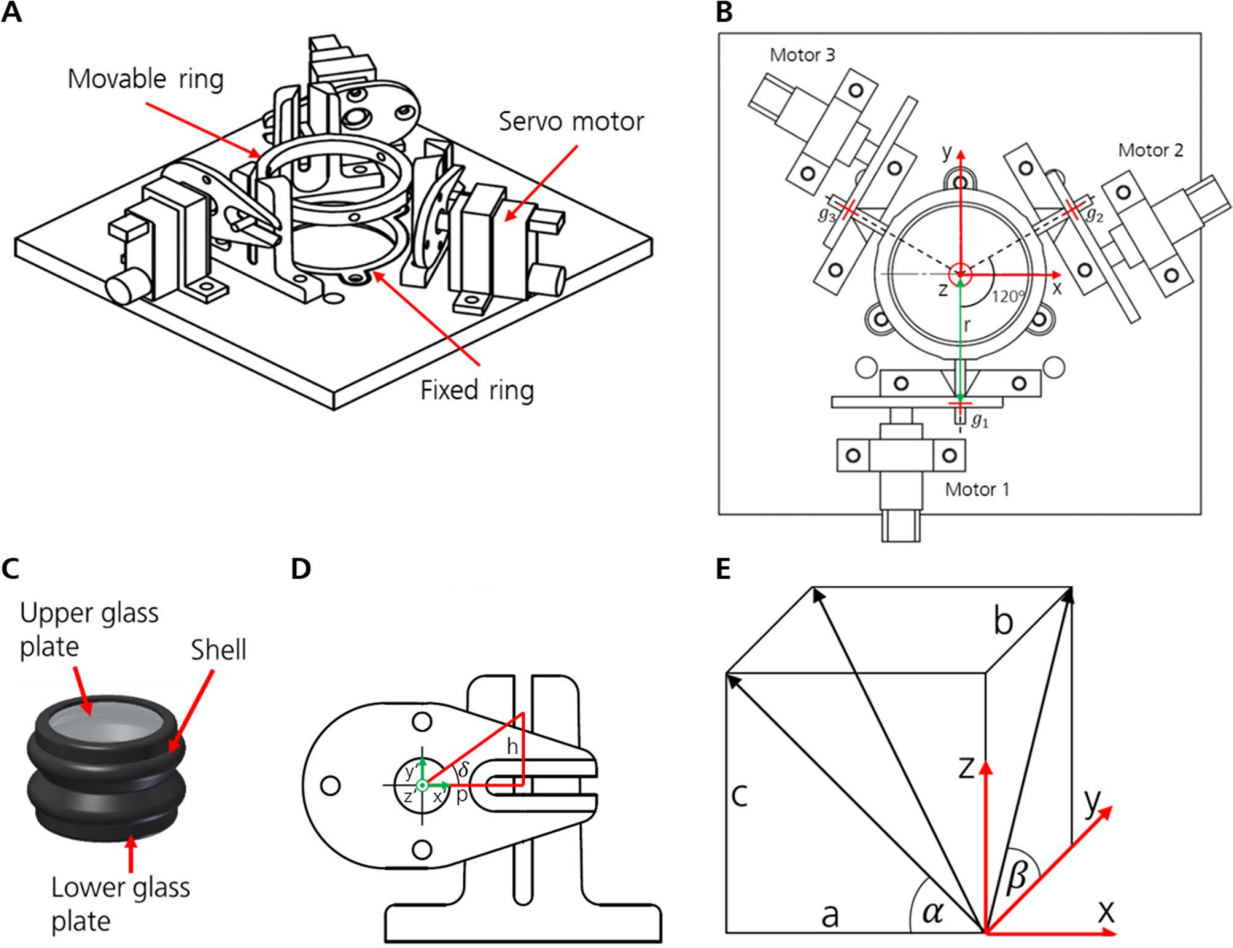
Prism design, actuation, and geometric relation. (**A**) An arrangement of the servos and rings that hold the prism (upside down). (**B**) Kinematic of the adaptive prism. View from below. (**C**) Prism design. (**D**) Kinematic model of one servo bearing. Front view. (**E**) Relationship between angles and coordinates that describe the glass plate with respect to the XY plane.

The adaptive prism was developed specifically for this application. An elastic skin and two glass plates form a closed volume into which approximately 10 ml of liquid medium is filled (Fig. 3C). The two cylindrical glasses have a diameter of 25.4 mm, a clear aperture of 22.86 mm, and a thickness of 1 mm. Both are anti-reflection coated with BBAR for visible light from 425 to 700 nm. Water was chosen as a liquid medium because of its ease of handling and compatibility with other materials. It has about the same refractive index as the cell nutrition solution (both n ≈ 1.333). Refraction at the glass plates’ surfaces (n ≈ 1.5) is neglected due to their small thickness.

The shell is manufactured by casting the soft-elastic polyurea casting compound GM 900-1 with a thickness of 1 mm. After filling, the glass planes were glued in place to form a seal. The elastic material allows inclination angles around the center point of the lower glass plate of up to 30°. Due to kinematic relations between the prism and servos, actual inclination angles are limited to approximately 20°.

The prism is mounted into an actuator unit. The upper glass plate is held horizontally, while the lower plate can be tilted by three servo motors. An overview of the unit is shown in Fig. 3A, while the exact servo motor positions in relation to the glass plate is depicted in Fig. 3B.

The rotation angle for each servo motor, called ${\gamma }_{1}$, ${\gamma }_{2}$, and ${\gamma }_{3}$, can be controlled to move to a desired glass plate angle. The lower glass plate plane is expressed with angles $\alpha$ and $\beta$, describing the rotation around the y-axis and x-axis, respectively (depicted in Fig. 3E).

To obtain the resulting servo angles from the glass plate’s angles, first the height of the levers at positions ${g}_{i}$ (with $i$ being the index of the motor) must be determined. Therefore, the lower glass plate of the prism is treated as a plane that can be described by the equation

1 $$E:=\left(\left(\begin{array}{c}x\\ y\\ z\end{array}\right)-\left(\begin{array}{c}0\\ 0\\ 0\end{array}\right)\right)\cdot \left(\begin{array}{c}a\\ b\\ c\end{array}\right)=0.$$

The position $\left(x, y, z\right)$ describes the coordinate of a point of the plane, which is fixed at the center of the coordinate system, therefore having $\left(0, 0, 0\right)$ as a plane base. The values $a$, $b$, and $c$ must be derived from the rotation angles of the plane, $\alpha$ and $\beta$, which represent the angles around the $y$ and $x$ axes, respectively. The plane can be described by two vectors on the $xy$ and $yz$ planes, called $\overrightarrow v$ and $\overrightarrow w$, respectively:

2 $$\overrightarrow {v} =\left(\begin{array}{c}1\\ 0\\ tan \;\alpha \end{array}\right),$$

3 $$\overrightarrow w=\left(\begin{array}{c}0\\ 1\\ tan \;\beta \end{array}\right).$$

The normal vector to the plane $E, \overrightarrow n = (a,b,c),$ can be derived from these two vectors using the cross product:

4 $$\overrightarrow n=\left(\begin{array}{c}a\\ b\\ c \end{array}\right)= \overrightarrow v \times \overrightarrow w.$$

Inserting Eqs. (2) and (3) into (4) equals

5 $$\left(\begin{array}{c}a\\ b\\ c \end{array}\right)=\left(\begin{array}{c}- tan\;\alpha\\ - tan\;\beta\\ 1 \end{array}\right).$$

The motors change the tilt angle of the prism’s glass plate ($\alpha$ and $\beta$) by raising and lowering the respective lever at the points of action, which are labeled with ${g}_{1}$, ${g}_{2}$, and ${g}_{3}$, respectively. The distance between the prism’s center and the points of action, labeled $r$, is 23 mm. The servo bearings’ positions concerning the height of the lever ${h}_{i}$ (see Fig. 3D) are described as follows:

6 $${\text{Motor }}\,1:{ }g_{1} { := }\left( {\begin{array}{*{20}c} 0 \\ { - r} \\ 0 \\ \end{array} } \right) + h_{1} \left( {\begin{array}{*{20}c} 0 \\ 0 \\ 1 \\ \end{array} } \right),$$

7 $${\text{Motor}}\,{ }2:{ }g_{2} { := }\left( {\begin{array}{*{20}c} {\cos 30^\circ \cdot r} \\ {\sin 30^\circ \cdot r} \\ 0 \\ \end{array} } \right) + h_{2} \left( {\begin{array}{*{20}c} 0 \\ 0 \\ 1 \\ \end{array} } \right),$$

8 $${\text{Motor }}\,3:{ }g_{3} { := }\left( {\begin{array}{*{20}c} { - \cos 30^\circ \cdot r} \\ {\sin 30^\circ \cdot r} \\ 0 \\ \end{array} } \right) + h_{3} \left( {\begin{array}{*{20}c} 0 \\ 0 \\ 1 \\ \end{array} } \right).$$

By inserting into the plane Eq. (1), the linear displacement can be calculated as follow:

9 $$\begin{aligned} {\text{Motor 1}}:\,\, & 0 \cdot a + \left( { - r} \right)b + \left( {0 + h_{1} } \right)c = 0 \\ & \quad \to h_{1} = r\frac{b}{c} \\ \end{aligned}$$

10 $$\begin{aligned} {\text{Motor 2}}:\,\, & \left( {\cos 30^\circ \cdot r} \right)a + \left( {\sin 30^\circ \cdot r} \right)b + \left( {0 + h_{2} } \right)c = 0 \\ & \quad \to h_{2} = - \frac{\cos 30^\circ ra + \sin 30^\circ rb}{c} \\ \end{aligned}$$

11 $$\begin{aligned} {\text{Motor 3}}:\,\, & \left( { - \cos 30^\circ \cdot r} \right)a + \left( {\sin 30^\circ \cdot r} \right)b + \left( {0 + h_{c} } \right)c = 0 \\ & \quad \to h_{3} = \frac{\cos 30^\circ ra - \sin 30^\circ rb}{c} \\ \end{aligned}$$

The rotation of the servo motors ${\delta }_{i}$ is converted into a vertical translation of levers ${h}_{i}$ using the known servo lever length $p$ (see servo bearing shown schematically Fig. 3D). The servo angle ${\delta }_{i}$ can be calculated using trigonometry:

12 $${\delta }_{i}=\mathrm{arctan}\frac{{h}_{i}}{p}.$$

### Adapter

**Figure 4:**
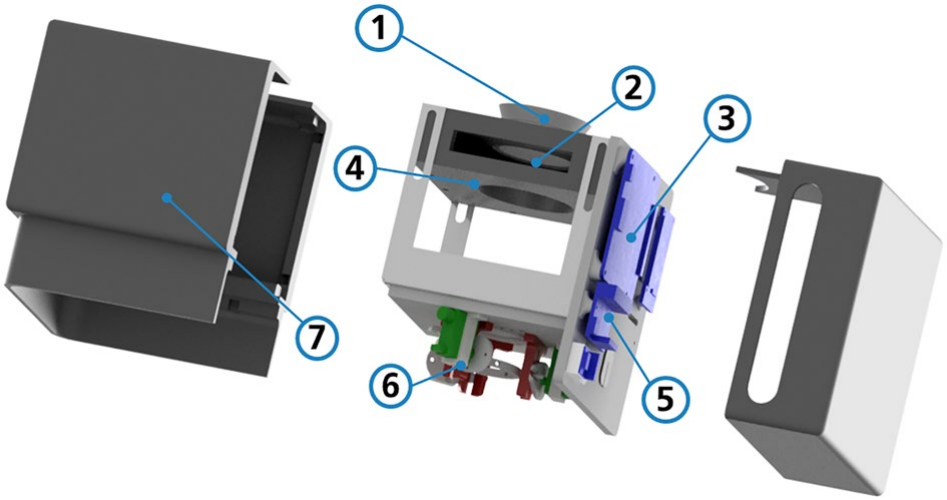
Optical components adapter. (1) Microscope mounting (2) LCD holder (3) LCD driver (4) Condenser lens mount (5) Motor driver (6) Adaptable prism (7) Housing. The light enters the adapter from the microscope mounting and leaves it after the adaptable prism.

A compact adapter, shown in Fig. 4, was designed to integrate the components into the optical path of the microscope. It consists of an LCD, a condenser lens, an adaptive prism, and multiple controllers. It is protected by a 3D-printed housing. Necessary drivers and controllers are already integrated into the adapter.

The adapter is designed to be mounted in place of a condenser unit in commercially available microscopes. Its compact design allows ample space between it and the microscope stage, facilitating the easy placement of MTPs.

### Automation

**Figure 5:**
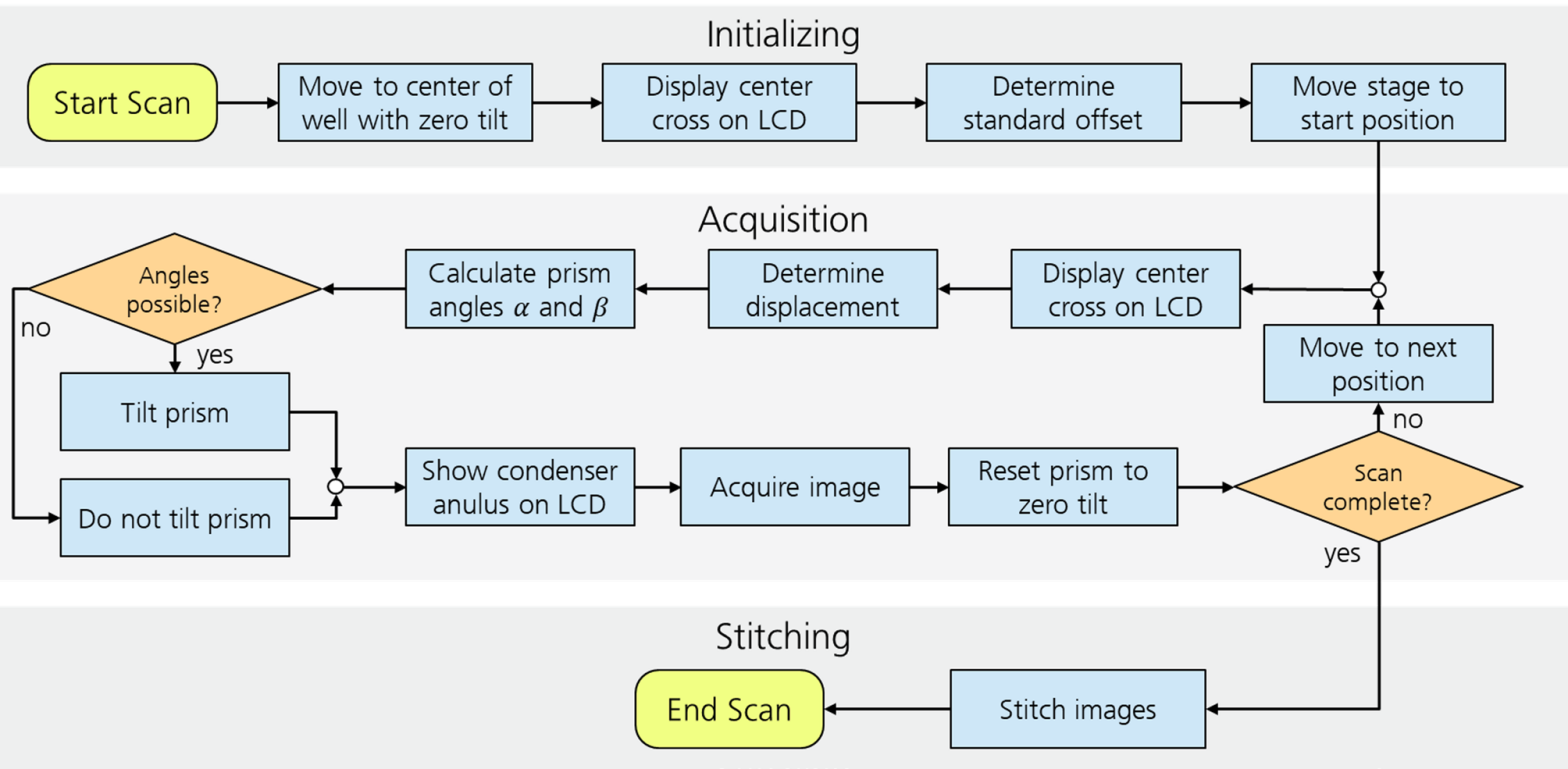
Flowchart of the image acquisition process. The process can be divided into three parts: Initialization, acquisition, and stitching. Initialization and stitching are only done once, at the beginning and end of the process, respectively. The acquisition process is repeated for every image.

The image acquisition process of a whole well was fully automated and schematically shown in Fig. 5. An initialization is carried out at the beginning. During this process, the microscope stage moves to the center of the well, and the LCD displays a cross. Then an image is taken by the secondary camera and, since no refraction should disturb the light in the middle of the well, the cross is supposed to appear in the center of the camera image undistorted. The exact position of the cross’ center is saved as offset $o$ for all following images. A cross is used as a shape because its center point can be determined more precisely than the ring which is used later.

After initialization, images are taken one by one. Initially, the prism is set vertically to zero tilt. After moving the microscope stage to the next position, a cross is again displayed on the LCD, and the location $l$ of the cross is determined like in the initialization.

The displacement $d$ is then calculated by subtracting the offset $o$ from the location $l$. It can be broken down into the displacements ${d}_{x}$ in x-direction and ${d}_{y}$ in y-direction. Empirical studies have shown that a linear correlation between prism angles $\alpha ({d}_{x})$ and $\beta ({d}_{y})$ and displacement $d$ exists for all angles the actuator can reach. Therefore, the prism angles are defined by the formulas

13 $$\alpha ={c}_{1}{d}_{x},$$

14 $$\beta ={c}_{2}{d}_{y},$$

with ${c}_{1}$ and ${c}_{2}$ being determined empirically. The resulting servo motor angles ${\delta }_{i}(\alpha ,\beta )$ are calculated using Eqs. (1) to (12).

The prism is then tilted accordingly. If the calculated angles are unreachable (for $\alpha , \beta >20^\circ$), the servos move to a neutral position and phase contrast is not achieved.

Thereafter, the LCD shows a condenser annulus with a displacement that matches the prism angles. The displacement was also determined empirically to match the prism rotation.

Having set the optimal parameters, the image is taken. Afterwards, the prism is reset to zero tilt, and the stage moves to the next position, and the process is then repeated. After taking the last image, all images are stitched together into one overall picture.

### Experiment design

To validate the effectiveness of the new technology, experiments were performed using the new setup and compared with results obtained using regular phase contrast microscopy. All measurements were carried out with the same microscope, with the adaptive components disabled during reference measurements.

The experiments were carried out with 6-, 12-, 24-, and 96-well-plates. For each set of measurements, the same cell populated MTP has been used. A focal plane was manually determined before the first acquisition and then held static throughout the process. The samples were first imaged with standard phase contrast and afterwards with the use of adaptive components. For both sets of images, sample images and Bertrand lens images were saved and then evaluated, respectively.

After the acquisition, the observable area was determined for each image. Phase contrast conditions were assumed to be where the background was darker than at the edge of the images and cells were surrounded by a ‘halo’, as proposed by Otaki^23^. The phase contrast ratio was calculated by dividing the phase contrast area by the area of the whole well.

## Results

**Figure 6:**
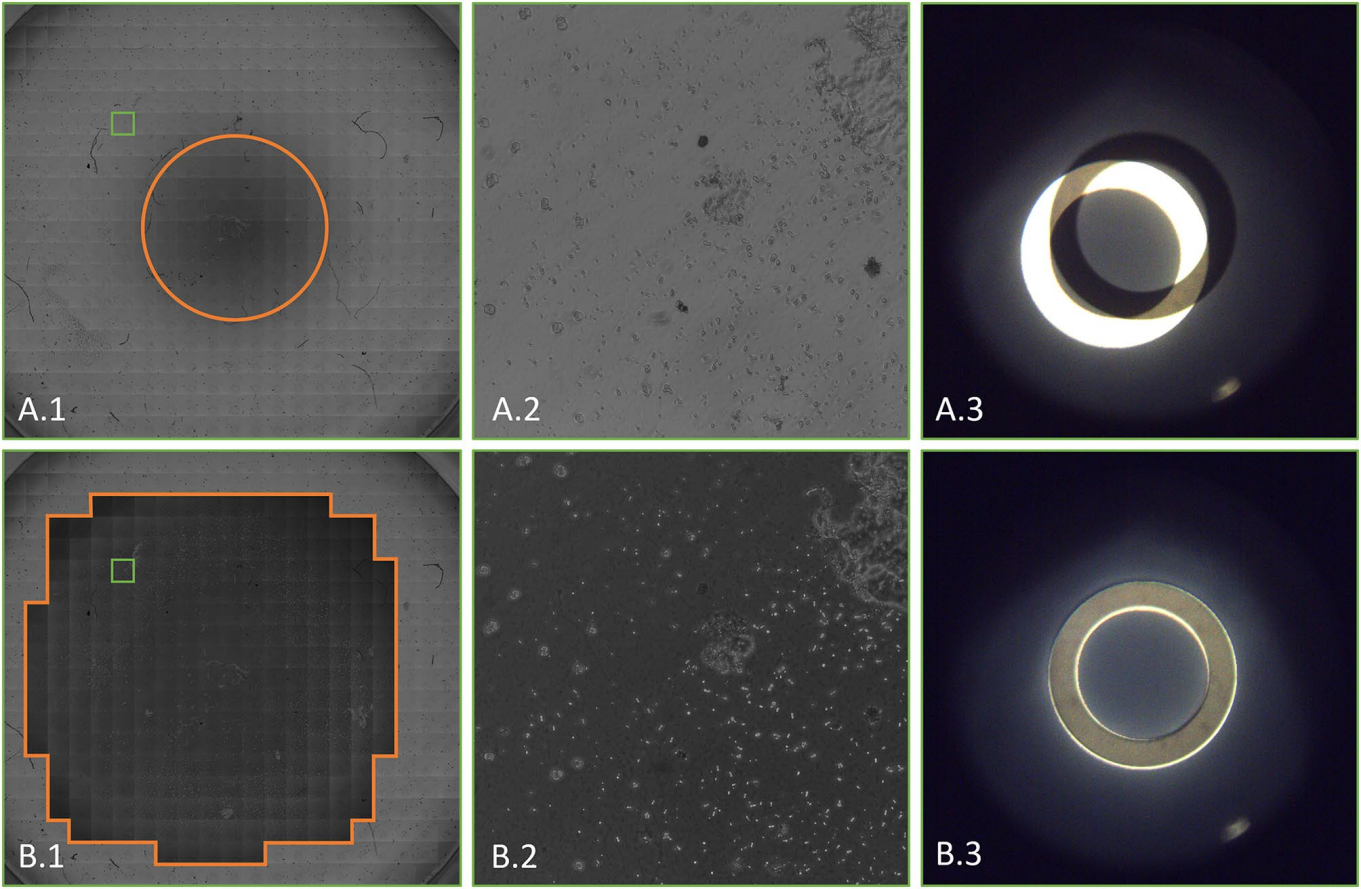
Comparison of standard (**A.1**–**A.3**) and adaptive (**B.1**–**B.3**) phase contrast microscopy images of one well in a 24-well MTP, 10x magnification. (**A.1/B.1**) Stitched images of the whole well. Phase contrast conditions are present in areas with a dark background. For clarification, they are outlined in orange. The images highlighted with a green box are shown in (**A.2/B.2**), respectively. (**A.2**) No phase contrast conditions are established. (**A.3**) The phase ring and condenser annulus do not align at the position of the image in (**A.2**). (**B.2**) Phase contrast conditions apply to the whole image. (**B.3**) The phase ring and condenser annulus do align at the position of the image in (**B.2**).

The difference in phase contrast area between the two methods is presented for a 24-well MTP in Fig. 6. Images A.2 and B.2 display the same location within the well using different methods. This clearly shows that the phase contrast range is further increased toward the edge by the adaptive method. This is confirmed as well by the superposition of phase rings in Fig. 6A.3 and B.3.

Furthermore, there is a sharp edge between phase contrast and non-phase contrast conditions in the adaptive approach. It is caused by the correction mechanism that stops once the liquid angle becomes uncorrectable, so either phase contrast conditions are established, or completely missed.

**Figure 7:**
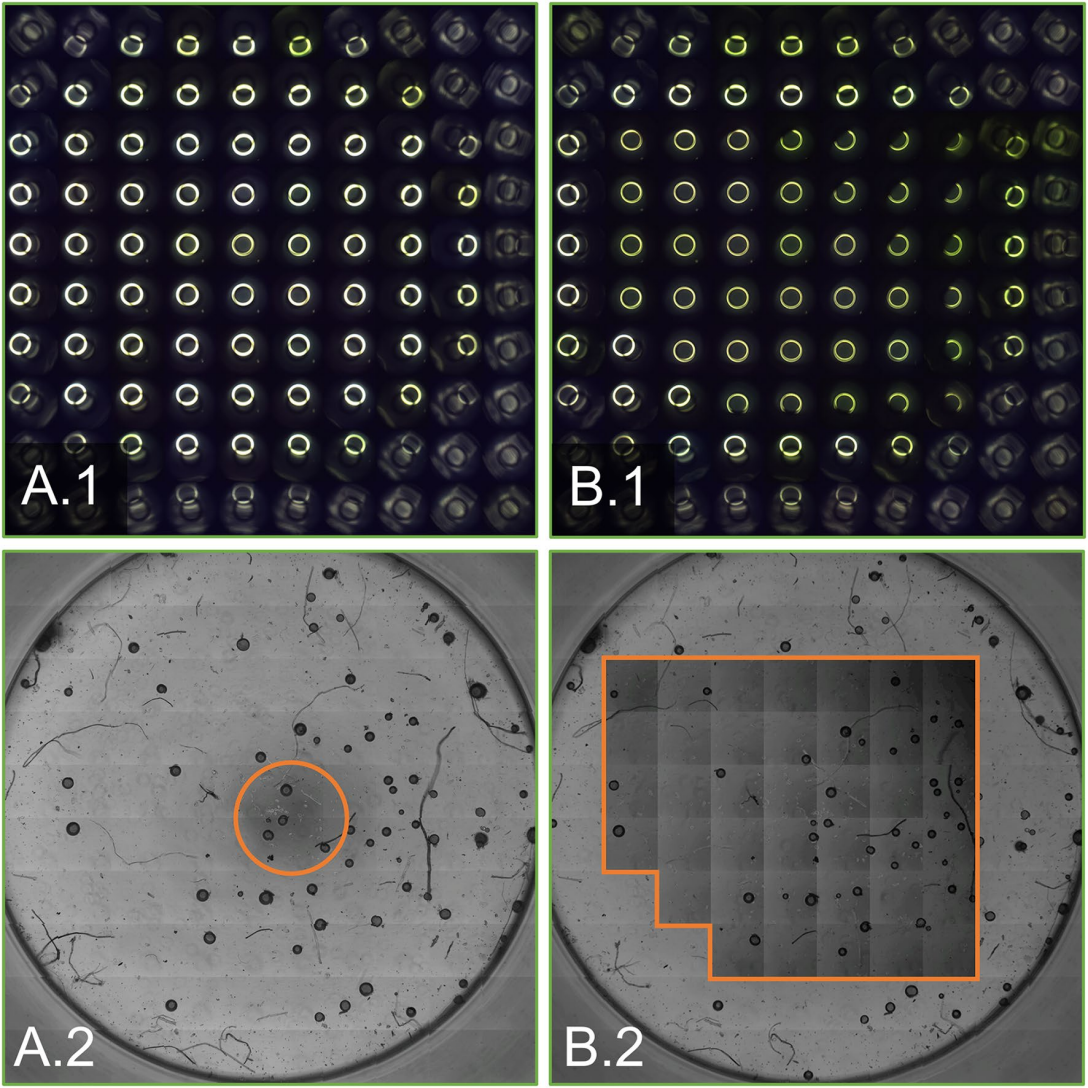
Comparison of the phase contrast images in one well of a 96-well MTP, 10x magnification. (**A**) Standard phase contrast. (**B**) Adaptive phase contrast. (**A.1/B.1**) Condenser annulus and phase ring alignment (**A.2/B.2**) Phase contrast images. Phase contrast areas are outlined in orange.

Another indicator of phase contrast conditions that are met in one image is the superposition of the condenser annulus and phase ring. Figure 7A.1,B.1 compares the superposition in every single image and the resulting phase contrast image for a 96-well-plate. The corresponding phase contrast images with the observable area highlighted is presented in Fig. 7A.2,B.2. The superposition images show that the difference in phase ring overlap is mostly prevalent close to the well's periphery, which is also visible in the phase contrast image.

Similar to the previously described experiments, the procedure has been repeated for other well-plates. Table 1 summarizes the experimental results for all investigated well sizes.

**Table 1:** Comparison between areas observable in phase contrast between standard and phase contrast methods. *MTP* Micro titer plate, *M* Magnification, *T* Imaging time per well, *A*_*Well*_ Area of one well, *A*_*sPC*_ Area observable in standard phase contrast, *rA*_*sPC*_ Relative phase contrast area A_sPC_/A_Well_, *A*_*aPC*_ Area observable in adaptive phase contrast, *rA*_*aPC*_ Relative phase contrast area A_aPC_/A_Well_, *R* Area ratio A_aPC_/A_sPC_.

MTP	M	T (min)	${A}_{Well}$ (mm^2^)	${A}_{sPC}$ (mm^2^)	${rA}_{sPC}$(%)	${A}_{aPC}$ (mm^2^)	${rA}_{aPC}$ (%)	R
6-Well	4x	43	940.2	235.1	25.0	432.5	46	1.84
12-Well	4x	22	394.1	33.5	8.5	90.6	23	2.78
24-Well	10x	65	206.1	17.7	8.6	86.6	42	3.98
96-Well	10x	17	36.3	0.84	2.3	10.2	28	12.15

The acquisition time for one image is approximately 5 s. The total process time depends on the number of shots needed to image a whole well and are listed in Table 1.

## Discussion

As shown in Table 1, the relative increase in observable area is significant for all examined well sizes. It ranges from almost doubling for 6-well-plates to more than 12-fold for 96-well MTPs. The increase in observable area is largest for smaller well sizes because the relative area that can be imaged with phase contrast decreases with the well diameter. The smallest wells in the experiments were those of 96-well-plates. It is not clear if the trend continues for even smaller wells because the observable area of wells of 96-well-plates is already only 2.3% and might not further decrease significantly. Moreover, smaller formats like 384-well-plates usually use rectangular wells instead of the round ones seen in larger formats. Yet, due to the adaptation approach, it is assumed that phase contrast conditions can also be enhanced for noncircular wells.

As presented in “Competing approaches”, a multitude of meniscus compensation approaches already exist. However, most of them are designed to work with one specific cell culture medium under pre-defined conditions. Thus, they are not robust under changing circumstances or with different mediums. Moreover, they require special microtiter plates, lids, or inserts that are significantly more expensive than standard microtiter plates. Microtiter plates are usually used in large quantities and are designed as disposable products, so the use of cost-effective standard products is advantageous.

Adaptive phase contrast microscopy offers a flexible approach to compensate for the meniscus effect that moreover works with conventional laboratory hardware. The technology exhibits several advantages over competing approaches, as evidenced by experimental results. Specifically, it enables a significant increase in the observable area in phase contrast. Due to its adaptability and flexibility, it can be used with different liquids with varying refractive indices. It does not even require MTPs but is applicable for all liquid filled vessels that fit on inverse microscopes. Because the phase contrast adapter can simply be installed in a regular inverse microscope, most standard functions are still available while providing an increased observable area with phase contrast microscopy. Furthermore, this approach also offers a viable non-invasive alternative to fluorescence microscopy.

These advantages also apply to Douglas’ approach which only uses the digital condenser annulus^18^. However, this alone is not sufficient to compensate for refraction close to the edge, especially when the liquid surface angle is steep. It can only compensate for the light beams’ lateral displacement $\Delta$, but not the angular rotation $\varphi$.

All images for one image series were always taken at the same focal plane. To improve acuity, future works that build on this technology should incorporate a focus correction mechanism. Yet, the current setup was sufficient to create expressive images as presented above.

One drawback of the presented approach is the slow image acquisition. Since an increasing number of laboratories become automated and require higher throughput, a fast acquisition process is necessary^24^. One approach to speed up the adaptive phase contrast process would be to acquire images during sample movement, which has already been shown for regular phase contrast microscopy^25^. It is planned to assess this approach in a future research project.

## Conclusion

Adaptive phase contrast microscopy makes it possible to significantly increase the area in which phase contrast microscopy is applicable. Quantifiable studies were performed using microtiter plates. The observable area was measured and compared to conventional phase contrast microscopy. A significant increase in the observable area was shown for all cases, with a larger effect on smaller wells due to a more pronounced meniscus effect. For 96-well-plates, the usable area is increased more than 12-fold, from about 2.3% of the total well area to about 28%, thus making phase contrast microscopy a viable option for non-invasive scanning of cell cultures.

Moreover, the solution can be fully automated so that integration in an automatic process is possible. Contrary to other methods to compensate for the meniscus effect, no special laboratory hardware is required, but standard microplates can be used.

Due to these reasons, the presented approach has the potential to extend the application area of phase contrast microscopy greatly, especially if the imaging time can be reduced.

## Data Availability

The authors confirm that the image data supporting the findings of this study are available within the article. The experimental setup has been built at Fraunhofer IPT and is described in the article. The control software is the intellectual property of Fraunhofer-Gesellschaft and therefore not eligible for publication.
