# Author Correction: Adaptive phase contrast microscopy to compensate for the meniscus effect

**DOI:** 10.1038/s41598-023-47733-1

**Published:** 2023-11-30

**Authors:** Florian Nienhaus, Tobias Piotrowski, Bastian Nießing, Niels König, Robert H. Schmitt

**Affiliations:** 1 Fraunhofer Institute for Production Technology IPT, Aachen, Germany; 2 WZL | RWTH Aachen University, Aachen, Germany

Correction to: *Scientific Reports* 10.1038/s41598-023-32917-6, published online 08 April 2023

The original version of this Article contained errors in Equations (2) - (5), in the Methods section, under the subheading ‘Adaptive prism’. The derivation of the normal vector of the plane equation for the lower glass plate was faulty. Equations (2) - (5) have now been corrected to accurately describe the relationship between the angles α and β and the plane $E$.

Consequently,

The values $a$, $b$, and $c$ must be derived from the rotation angles of the plane, $\alpha$ and $\beta$, considered as two angles in a cuboid with diagonal $\left|v\right|=1$.

According to the laws of cosines and sines, as well as the Pythagorean theorem, three formulas apply, so that the values for $a$, $b$, and $c$ can be calculated. They are:

2 $${a}^{2}+{b}^{2}+{c}^{2}=\left|v\right|=1$$

3 $$a=\frac{c}{\mathrm{tan}\alpha }$$

4 $$b=\frac{c}{\mathrm{tan}\beta }$$

Inserting equations (3) and (4) into (2) equals

5 $$c=\sqrt{\frac{1}{1+\frac{1}{{\mathrm{tan}}^{2}\alpha }+\frac{1}{{\mathrm{tan}}^{2}\beta }} }.$$

now reads:

The values $a$, $b$, and $c$ must be derived from the rotation angles of the plane, $\alpha$ and $\beta$, which represent the angles around the $y$ and $x$ axes, respectively. The plane can be described by two vectors on the $xz$ and $yz$ planes, called  $\overrightarrow{v}$ and $\overrightarrow{w}$, respectively:

2 $$\overrightarrow{v}=\left(\begin{array}{c}1\\ 0\\ \mathrm{tan}\alpha \end{array}\right),$$

3 $$\overrightarrow{w}=\left(\begin{array}{c}0\\ 1\\ \mathrm{tan}\beta \end{array}\right).$$

The normal vector to the plane $E$, $\overrightarrow{n}=\left(a, b, c\right)$, can be derived from these two vectors using the cross product:

4 $$\overrightarrow{n}=\left(\begin{array}{c}a\\ b\\ c\end{array}\right)=\overrightarrow{v}\times \overrightarrow{w}.$$

Inserting equations (2) and (3) into (4) equals

5 $$\left(\begin{array}{c}a\\ b\\ c\end{array}\right)=\left(\begin{array}{c}-\mathrm{tan}\alpha \\ -\mathrm{tan}\beta \\ 1\end{array}\right).$$

In addition, Figure 3 has been updated as the norm of the vector is not relevant. The original Figure 3 and accompanying legend appear below.

**Figure 3:**
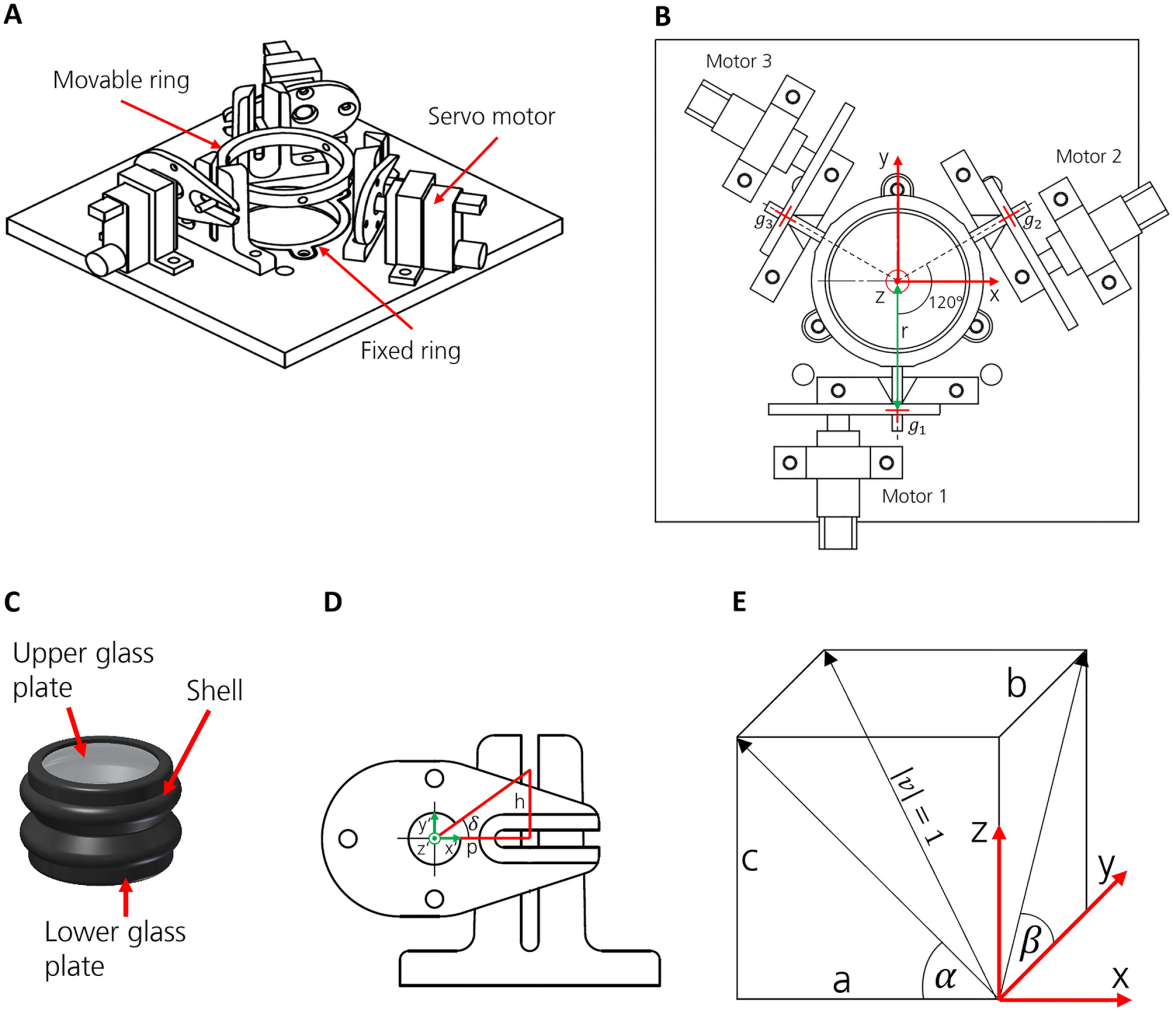
Prism design, actuation, and geometric relation. (**A**) An arrangement of the servos and rings that hold the prism (upside down). (**B**) Kinematic of the adaptive prism. View from below. (**C**) Prism design. (**D**) Kinematic model of one servo bearing. Front view. (**E**) Relationship between angles and coordinates that describe the glass plate with respect to the XY plane.

The original Article has been corrected.

